# Ophthalmological symptoms in a patient with reversible cerebral vasoconstriction syndrome: a case report

**DOI:** 10.1186/s13256-021-02746-0

**Published:** 2021-03-28

**Authors:** Rijo Hayashi, Shimmin Hayashi, Shigeki Machida

**Affiliations:** 1 Department of Ophthalmology, Saitama Medical Center, Dokkyo Medical University, 2-1-50 Minamikoshigaya, Koshigaya, Saitama 343-8555 Japan; 2 Lively Eye Clinic, 3-1-4 Asahicho, Soka, Saitama 340-0053 Japan

**Keywords:** Reversible cerebral vasoconstriction syndrome, Ophthalmological symptoms, Thunderclap headache, Postpartum, Homonymous hemianopsia, Exudative retinal detachment

## Abstract

**Background:**

Reversible cerebral vasoconstriction syndrome is characterized by severe headache with reversible segmental constriction of the cerebral arteries. We present details on a patient with reversible cerebral vasoconstriction syndrome who initially visited an ophthalmologist because of visual symptoms.

**Case presentation:**

A 34-year-old Japanese woman complained of sustained headache and insomnia starting 2 days after her first childbirth. In addition to the severe headache, a visual field defect was also observed 10 days later. Best corrected visual acuity at the initial visit was 20/20 and 20/25 for the right and left eye, respectively. Exudative retinal detachment was noted surrounding both optic heads. Visual field testing revealed left homonymous hemianopsia, while magnetic resonance imaging demonstrated the presence of edema and infarction of the bilateral basal nuclei and right occipital lobe. The homonymous hemianopsia and exudative retinal detachment recovered immediately after treatment with a free-radical scavenger and anticoagulant. There has been no recurrence of symptoms during the 4 years of follow-up.

**Conclusions:**

We report a case of reversible cerebral vasoconstriction syndrome with ophthalmological symptoms that were reversible, including serous retinal detachment and homonymous hemianopsia.

## Background

The incidence of stroke in the peripartum and postpartum periods has increased in recent years and might correspond to the observed increase in pregnancy-induced hypertension [1, 2]. There are several conditions associated with hypertension, including posterior reversible encephalopathy, reversible cerebral vasoconstriction syndrome, and cerebral venous sinus thrombosis, along with an increased risk of stroke and vascular dementia [1]. Reversible cerebral vasoconstriction syndrome (RCVS) is characterized by reversible constriction of cerebral arteries associated with severe headache [3, 4], which is referred to as thunderclap headache [5]. There are various precipitating factors, with the most common being postpartum [6] and exposure to vasoactive substances [4, 7]. Patients with RCVS usually visit emergency rooms because of the severe headache. However, postpartum patients with RCVS usually visit obstetricians. Visual deficits have only been reported in 29% of RCVS patients [8], and ophthalmological findings have yet to be reported in detail. In this report, we present ophthalmological findings for a patient with RCVS who visited our clinic because of visual field defects.

## Case presentation

A 34-year-old Japanese woman suffered from headache and insomnia starting 2 days after her first childbirth. During her pregnancy, she did not experience any complications, including pregnancy-induced hypertension. However, postpartum hypertension was noted (160–170 mmHg/90–100 mmHg). She visited our clinic complaining of a visual field defect that had appeared 8 days after her headache onset.

**Fig. 1:**
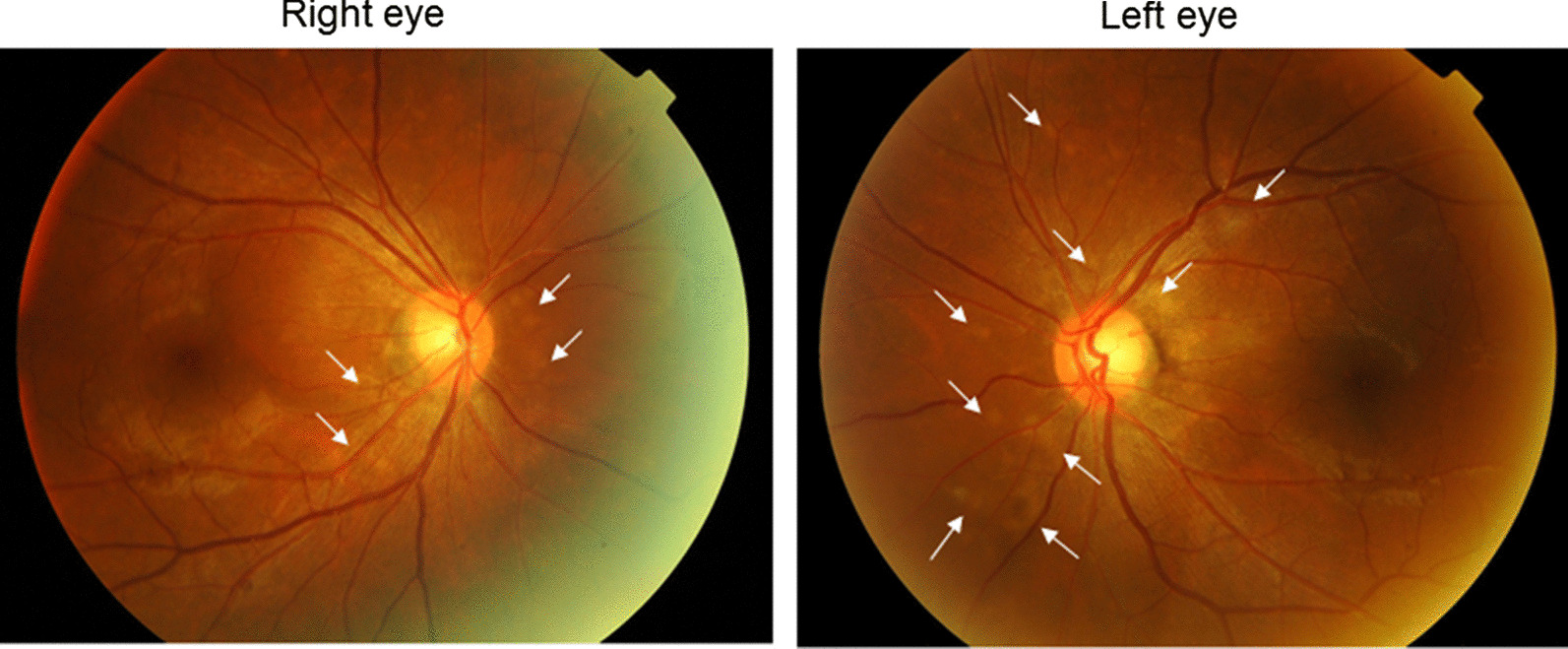
Fundus photograph at the first visit. Exudative edema was noticed surrounding both of the optic heads. Elschnig’s spots (*arrows*) and ischemic choroidal infarcts were also revealed

**Fig. 2:**
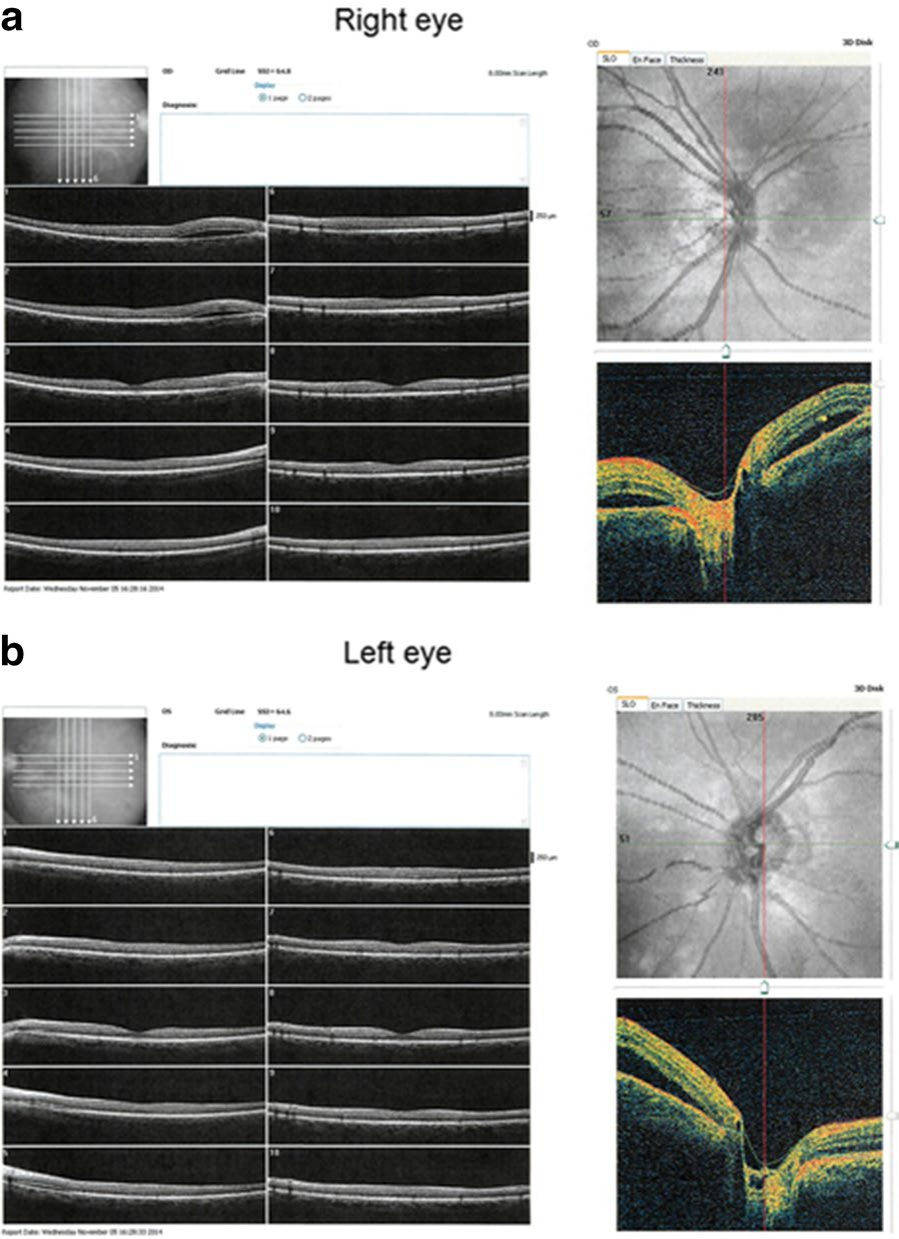
Optical coherence tomography examination near the optic head at the first visit. Exudative retinal detachments were associated with both optic heads and corresponded to the peripapillary exudative edema. **a** right eye, **b** left eye

**Fig. 3:**
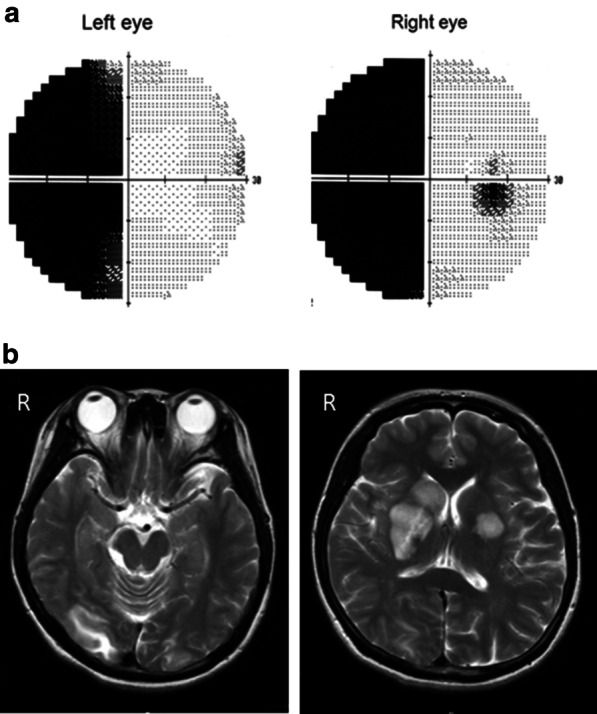
Visual field and magnetic resonance imaging before treatment. **a** Left homonymous hemianopsia was revealed. **b** MRI at 3 days after onset of the severe headache. T1-weighted-fluid-attenuated inversion recovery (T1-FLAIR) MRI revealed edema and infarction in the basal ganglions and right occipital lobe

Corrected visual acuities for the right and left eye were 20/20 and 20/25, respectively. Intraocular pressure in both eyes was 15 mmHg. The anterior segments and lenses appeared normal. Multiple yellowish dots in the deep retina were noted around the optic nerve head in both eyes (indicated by the arrows in Fig. 1), and resembled Elschnig’s spots and ischemic choroidal infarcts [9]. Optical coherence tomography (OCT) demonstrated exudative retinal detachments around the optic nerve head in both eyes (Fig. 2). Visual field testing conducted by standard automated perimetry showed left homonymous hemianopsia (Fig. 3a).

We referred the patient to a neurologist for neurological examinations regarding the headache and left homonymous hemianopsia. Results from serum biochemistry tests and a cerebrospinal fluid examination were normal. Magnetic resonance imaging (MRI) revealed edema in the basal ganglions and right occipital lobe (Fig. 3b).

**Fig. 4:**
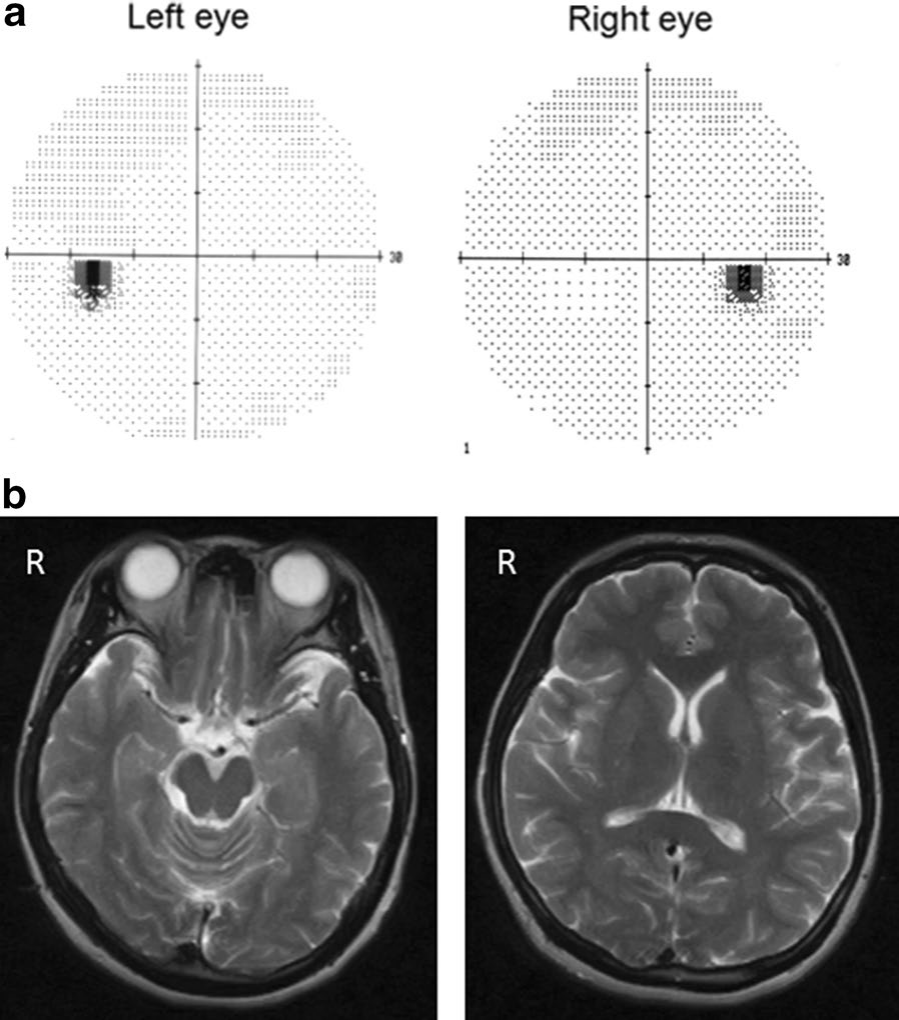
Visual field and magnetic resonance imaging after treatment. **a** Left homonymous hemianopsia improved at 2 days after infusion of a free-radical scavenger and anticoagulant. **b** MRI at 1 month after treatment. The lesions in the basal ganglions and occipital lobe were no longer visible in the follow-up MRI

**Fig. 5:**
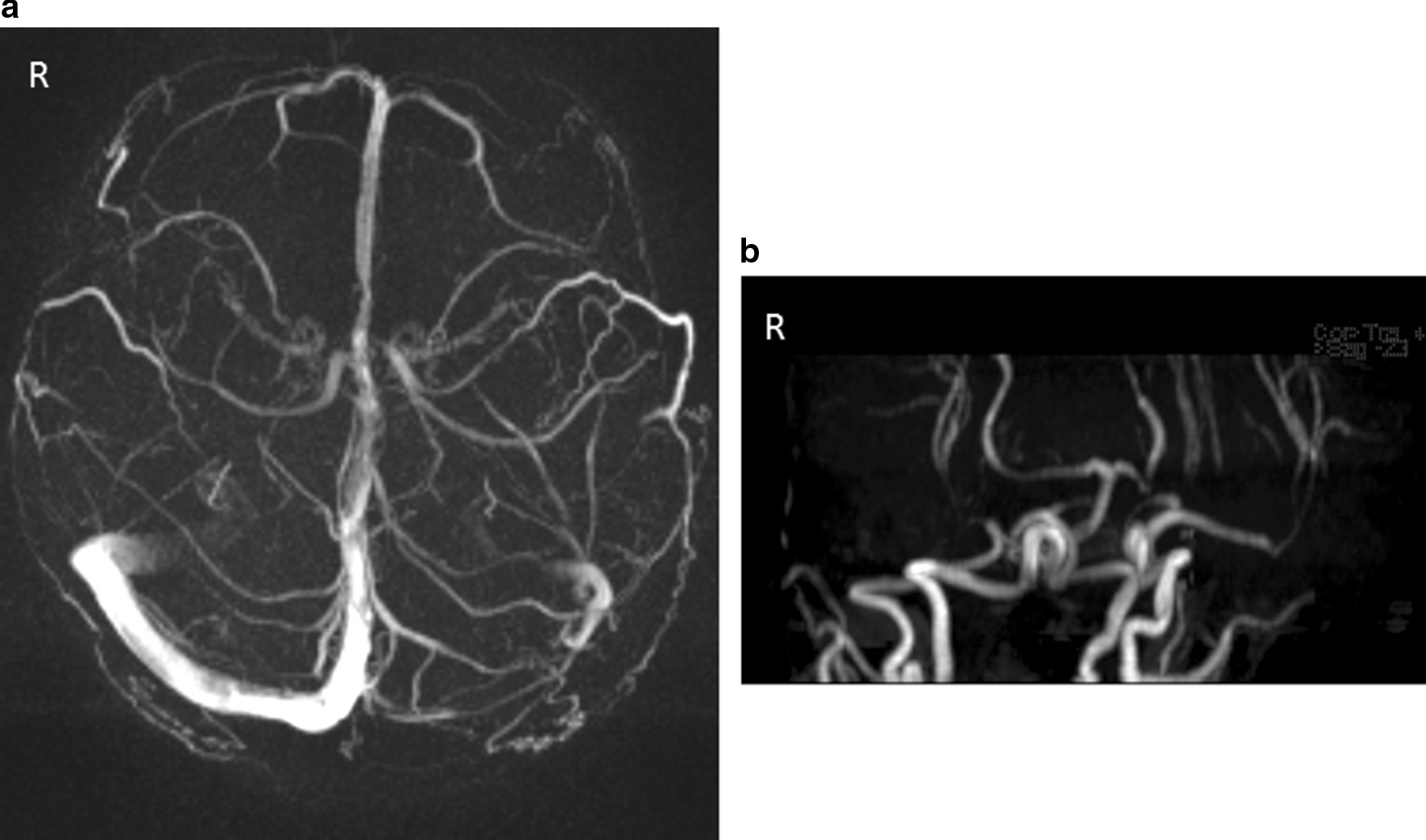
Angiography performed after improvement of homonymous hemianopsia. Normal angiography was observed in the patient following improvement of the homonymous hemianopsia. **a** Magnetic resonance venography. **b** Magnetic resonance angiography

**Fig. 6:**
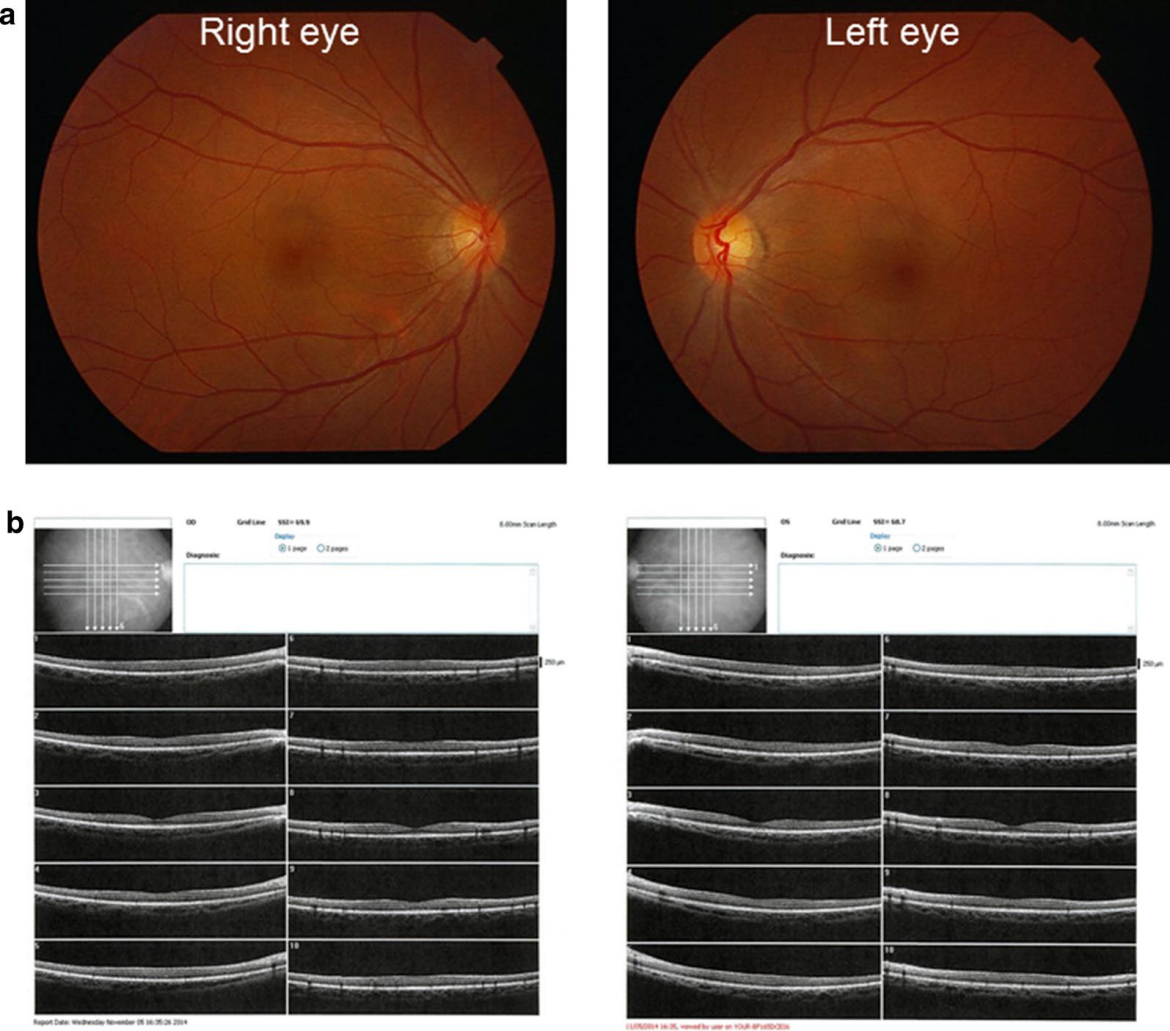
Fundus photograph and OCT at 1 month after treatment. **a** Exudative retinal detachments and exudative edema surrounding the optic heads were no longer visible in the images. **b** Exudative retinal detachment and edema were no longer visible in the images

At day 2 after starting treatments with an infusion of a free-radical scavenger (edaravone, 60 mg per day) and anticoagulant (heparin sodium, 10,000 U per day), the left homonymous hemianopsia disappeared (Fig. 4a) and MRI showed subsidence of the brain edema (Fig. 4b). The treatment was continued for 3 days, and the patient's symptoms improved. After improvement of the visual symptoms, cerebral angiography appeared normal (Fig. 5), and the yellowish dots in the deep retina (Fig. 6a) and exudative detachments around the optic nerve heads were no longer observed (Fig. 6b). There has been no recurrence of symptoms during the 4 years of follow-up.

The patient gave consent for her clinical details and clinical images to be published.

## Discussion

RCVS is an acute and self-limiting disease commonly accompanied by acute, severe headache. The most significant contributor to RCVS is being postpartum [10–12], with hypertension also reported to be a contributing factor [13]. The present patient suffered from both hypertension and severe headache at 2 days after her first childbirth. However, there was improvement in the headache and visual symptoms within 2 weeks. The clinical course of this patient was consistent with that for RCVS.

On cerebral angiography, RCVS can present as segmental narrowing and dilation of one or more arteries [4]. However, vasoconstriction in cerebral angiography is not essential in the diagnosis of RCVS, as the sensitivity has been reported to be only about 70% [14]. The normal angiography observed in RCVS patients may be due to the timing, as vasoconstriction may not be revealed during the 2 weeks after the initial clinical onset [15]. The normal cerebral angiography observed in our patient occurred after the improvement of symptoms, so the vasoconstriction could have possibly been in remission. The presence of hemorrhage or aneurysm should be ruled out for a diagnosis of RCVS [8, 16]. Neither hemorrhage nor aneurysm was found in our patient, who was correctly diagnosed with RCVS.

Because of visual deficits, the patient presented to our clinic instead of visiting either the emergency room or her obstetrician. Blurred vision has been previously reported to be one of the clinical manifestations of RCVS that is due to brain lesions [8, 10, 12, 13, 17]. Although homonymous hemianopsia was previously reported in a case examined using a confrontation visual field test [17], detailed ocular findings have not been reported in other prior RCVS cases. However, deterioration of visual acuity and visual field defects have been reported in patients with postpartum posterior reversible encephalopathy syndrome (PRES) [18–20], which shares several common clinical and radiologic features with RCVS [21]. Although PRES frequently exhibits a symmetric distribution of changes in the parietooccipital lobes with a moderate headache, these findings were not consistent with the results found in our patient. Vasoconstrictive involvement of the parietooccipital lobes might be a mechanism for severe visual deficits [22], and is one possible explanation for the homonymous hemianopsia in our patient.

Exudative retinal detachments have also been reported in PRES patients with hypertension [23] and in pregnancy [24]. Previous studies have reported finding constriction of choroidal arterioles in the acute ischemic phase of hypertension [25], followed by patchy filling of choroidal capillaries [26] and dysfunction of the retinal pigment epithelium [25], which induced exudative retinal detachment. The exudative retinal detachment found in our patient may also have been due to the same mechanism, with the presence of the Elschnig’s spots supporting the constriction of choroidal arterioles.

Excellent clinical outcome has been reported in 80% of patients [6], with fatalities reported in less than 1% of RCVS cases [8, 13, 16]. However, there has been a higher nonbenign outcome rate reported in postpartum RCVS, with death in 20% and residual deficits in 30% of the cases [27]. Thus, close observation of patients with postpartum RCVS is necessary. In addition, any abnormal ocular findings on diagnostic examinations should be considered potential visual defects associated with the initial symptoms of RCVS.

## Conclusions

In this study, we reported on a patient with a visual defect that was an initial symptom of RCVS. This is the first report to present ophthalmological findings for RCVS. Although RCVS is known to have a good prognosis, sometimes stroke or neurological sequelae can complicate the condition, and thus, close observation of these patients is necessary.

## Data Availability

The datasets used and/or analyzed during the current study are available from the corresponding author on reasonable request.

## Abbreviations

RCVS: Reversible cerebral vasoconstriction syndrome
OCT: Optical coherence tomography
MRI: Magnetic resonance imaging
PRES: Posterior reversible encephalopathy syndrome
